# Supplementary figures and images for: MCHM Acts as a Hydrotrope, Altering the Balance of Metals in Yeast
Source: Biol Trace Elem Res. 2019 Aug 7;195(1):260–71. doi: 10.1007/s12011-019-01850-z (PMC7150659; doi:10.1007/s12011-019-01850-z)

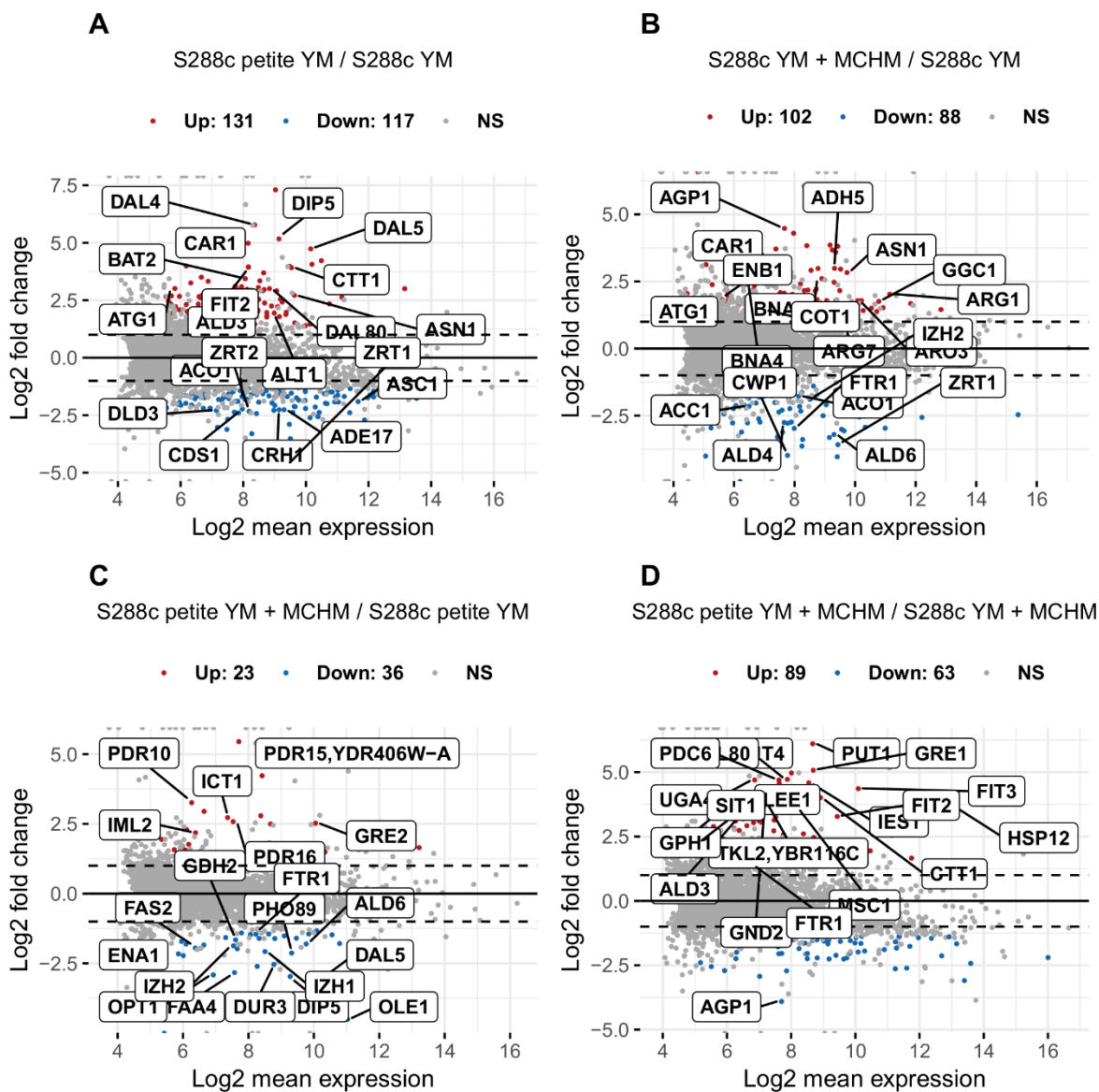

Supplemental Figure 1 Pupo 2018

Supplement: Supplementary file 1 — Scatter plots of log fold 2 comparisons of RNA-seq from grande (S96) and petite (S96) yeast grown in YM supplemented with lysine. Significantly up-regulated genes are labeled in red and significantly down-regulated genes are labeled in blue. (A) Scatter plots of log fold 2 comparisons of RNA-seq petite and grande yeast grown in YM. (B) Scatter plots of log fold 2 comparisons of RNA-seq grande yeast grown in YM and with 550 ppm MCHM. (C) Scatter plots of log fold 2 comparisons of RNA-seq from petite yeast grown in YM and with MCHM. (D) Scatter plots of log fold 2 comparisons of RNA-seq from grande and petite yeast grown in YM and with MCHM. (PDF 530 kb) [file 12011_2019_1850_MOESM1_ESM.pdf]

## Up-regulated Genes

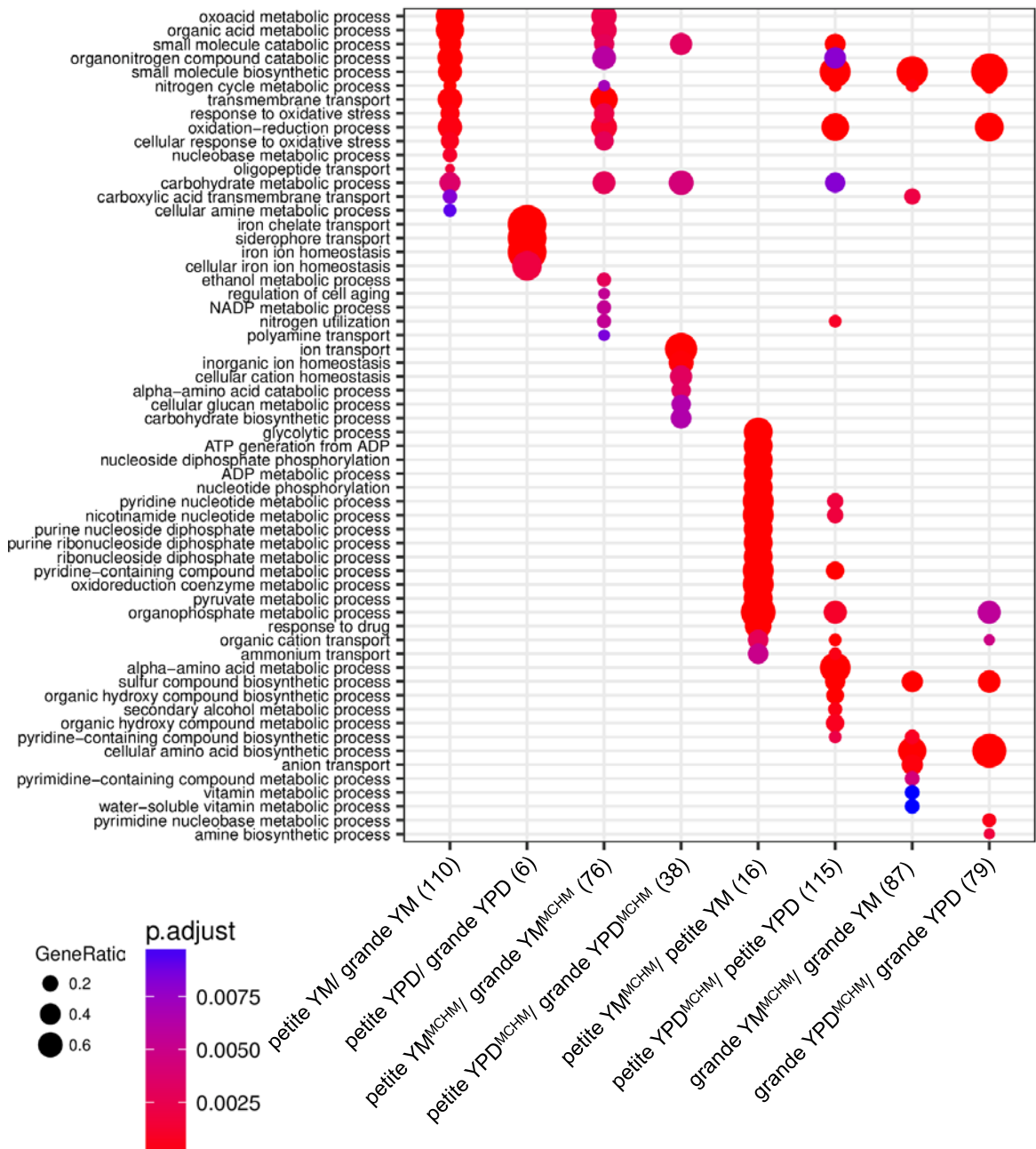

Supplemental Figure 2 Pupo 2018

Supplement: Supplementary file 2 — GO term analysis of genes up-regulated in all pairwise comparisons with single variable between S96 grande and S96 petite yeast grown in YM and YPD with MCHM added. The color scale of p adjust values are noted and the size of the circle notes gene ratio. The number of genes in each comparison is noted in parentheses. (PDF 338 kb) [file 12011_2019_1850_MOESM2_ESM.pdf]

## Down-regulated Genes

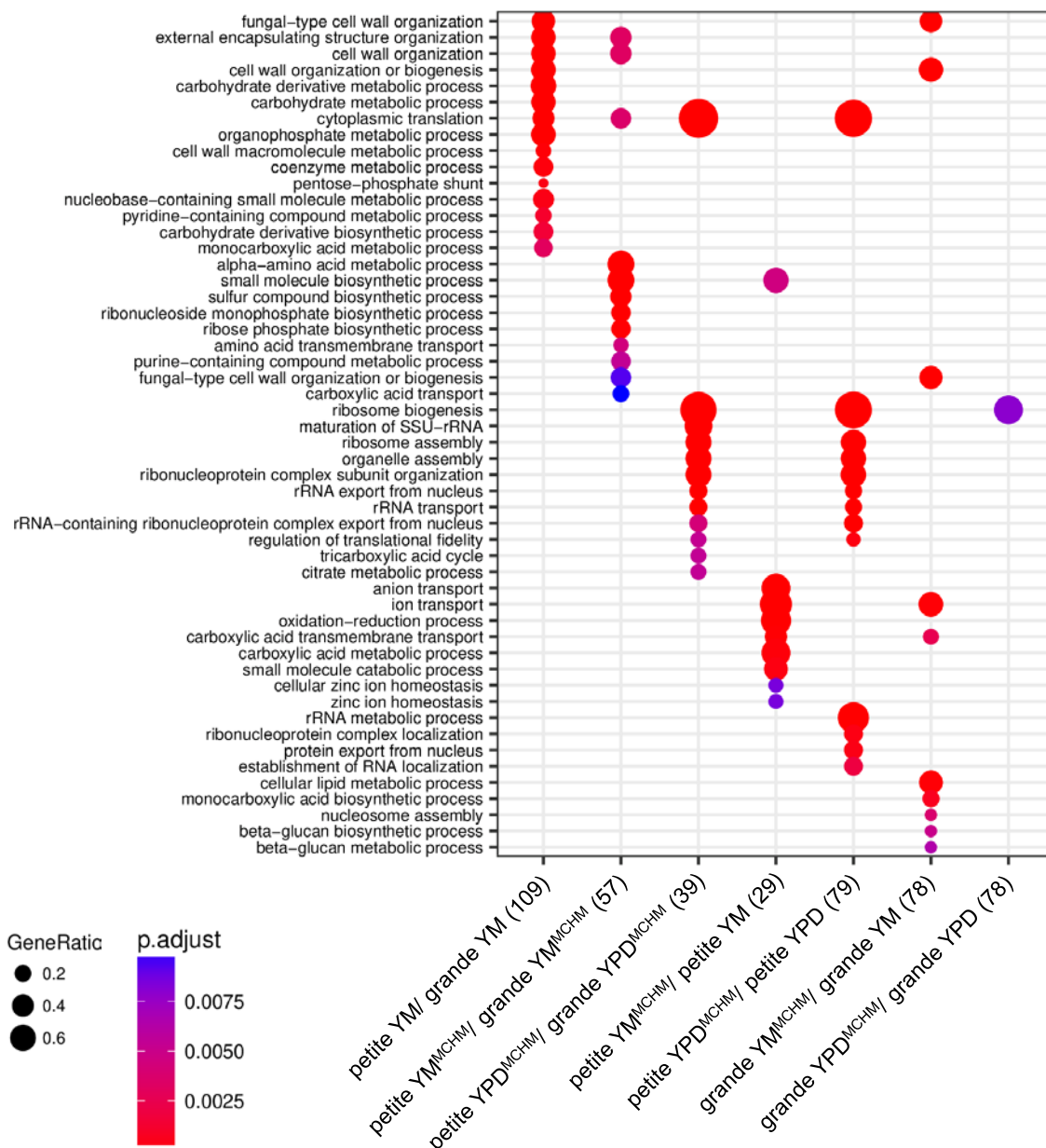

Supplemental Figure 3 Pupo 2018

Supplement: Supplementary file 3 — GO term analysis of genes down-regulated in all pairwise comparisons with single variable between S96 grande and S96 petite yeast grown in YM and YPD with MCHM added. The color scale of p adjust values are noted and the size of the circle notes gene ratio. The number of genes in each comparison is noted in parentheses. (PDF 299 kb) [file 12011_2019_1850_MOESM3_ESM.pdf]

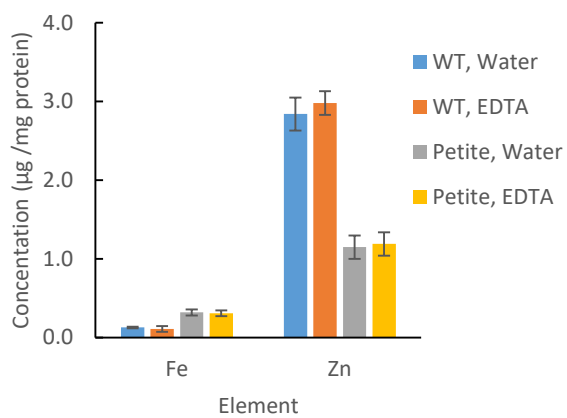

**Supplemental Figure 4 Pupo 2018**

Supplement: Supplementary file 4 — Levels of Fe and Zn in grande and petite S96 yeast washed with water and EDTA or with only water before metal extraction. Mean of four biological replicates are shown with standard error. (PDF 44 kb) [file 12011_2019_1850_MOESM4_ESM.pdf]

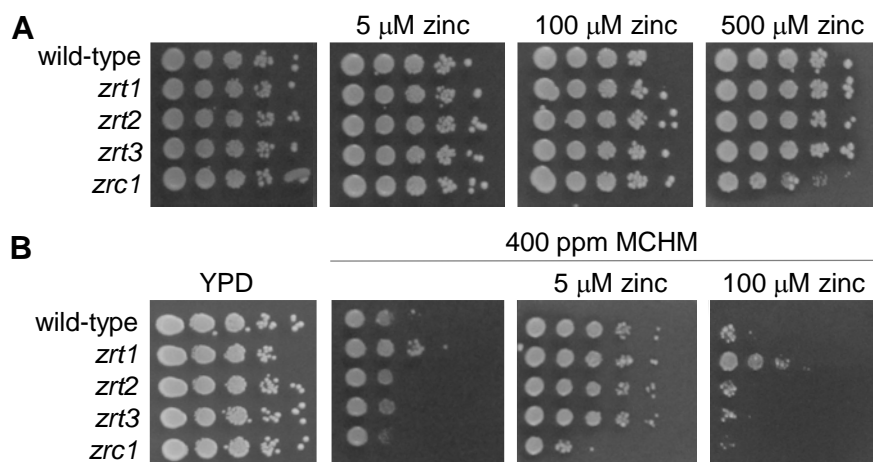

**Supplemental Figure 5 Pupo 2018**

Supplement: Supplementary file 5 — Role of zinc transporters in MCHM response with supplemented zinc. (A) Serial dilution of wild-type (BY4741) and zinc transporter knockout yeast were grown on YPD supplemented with increasing concentrations of zinc sulfate. (B) Serial dilution of wild-type (BY4741) and zinc transporter knockout yeast grown in 400 ppm MCHM on YPD supplemented with increasing concentrations of zinc sulfate. (PDF 140 kb) [file 12011_2019_1850_MOESM5_ESM.pdf]

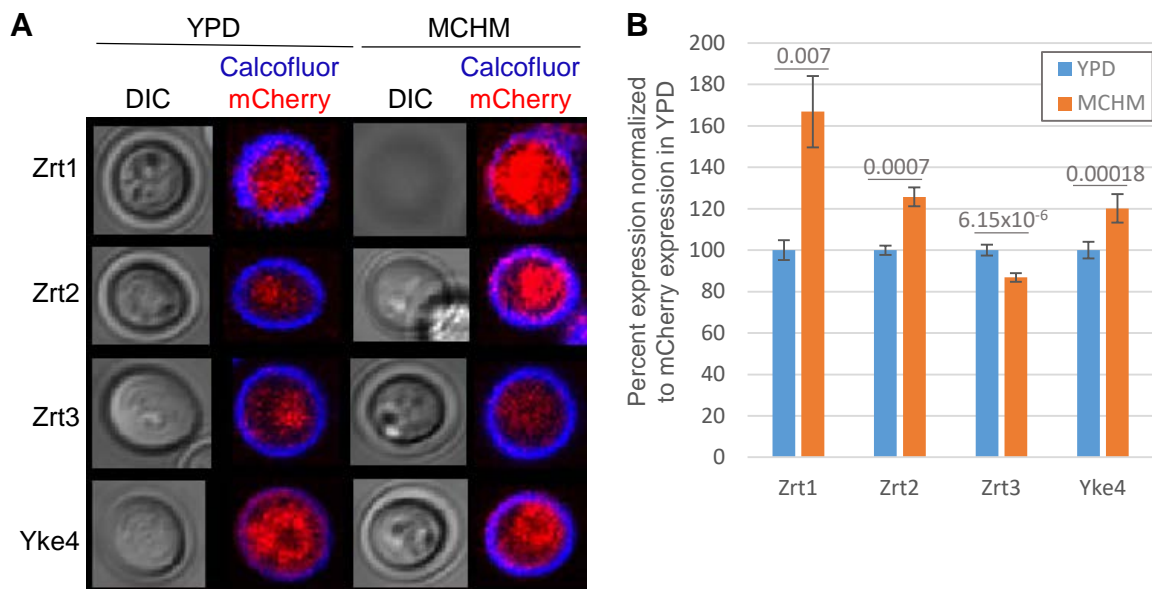

**Supplemental Figure 6 Pupo 2018**

Supplement: Supplementary file 6 — Levels of mCherry tagged Zrt1, Zrt2, Zrt3, and Yke4 proteins change with MCHM exposure. Yeast were exposed to 550 ppm of MCHM in YPD for 30 min. (A)The cell wall was stained with calcofluor white. (B) The mean fluorescence of mCherry normalized to untreated yeast for each protein. Quantification for 17–20 cells shown with standard error. (PDF 64 kb) [file 12011_2019_1850_MOESM6_ESM.pdf]
